# Iron infusion in pregnancy and dental dysplasia in children—is there a link?

**DOI:** 10.3389/fped.2025.1583241

**Published:** 2025-06-25

**Authors:** Gabriela Amstad, Tilo Burkhardt

**Affiliations:** Department of Obstetrics, University Hospital Zurich, Zurich, Switzerland

**Keywords:** iron infusion, mineralization, hypophosphatemia, phosphate, dental dysplasia

## Abstract

Some intravenous iron preparations cause hypophosphatemia mediated by increased fibroblast growth factor 23. This hypophosphatemia lasts for weeks or months and, when administered to pregnant women, could affect fetal tooth mineralization, which starts in the fourth month of pregnancy. The fetus requires increased calcium and phosphate levels to meet the increased demand for bone and tooth mineralization, development, and growth. As bone mineralization is a priority, calcium and phosphate deficiency could be compensated for by impaired primary and permanent tooth mineralization. Since there is an association between calcium and phosphate deficiency and dental dysplasia in X-linked hypophosphatemic rickets, we hypothesize a possible similar association between hypophosphatemia induced by intravenous iron infusion and dental dysplasia. As the long-term clinical impact of maternal hypophosphatemia on the fetus has not yet been investigated, studies are required to examine the effects of maternal hypophosphatemia on the fetus. Close cooperation between obstetricians, pediatric dentists, and pediatricians is essential to study the effect of hypophosphatemia induced by intravenous iron infusion on the primary and permanent tooth maturation and mineralization, growth, and development in children.

## Introduction

The administration of iron infusions to pregnant women is common, as it is very effective in one or two doses, with a low risk of allergic reactions and rare adverse side effects (1). One of the most common side effects is hypophosphatemia (2), which is usually mild without clinical symptoms or clinical consequence. But is it really so?

A normal calcium (Ca) and phosphate (P) content during pregnancy is necessary for normal mineralization of the child's bones and teeth. The normal serum P and Ca concentrations in fetus and children are significantly higher compared to adults (3). Therefore, even mild (asymptomatic) maternal hypophosphatemia may lead to severe fetal and neonatal hypophosphatemia. The relatively low P levels in neonates increase shortly after birth, likely associated with increased gluconeogenesis and endogenous P release, or secondary to a low glomerular filtration rate and reduced P excretion (4). The mean serum P level rises until the first week and then falls to levels corresponding to those in childhood (4). The prenatal and postnatal period is a vulnerable phase in the development and mineralization of the teeth due to mineralization of the second primary molars starting from fourth month of pregnancy, the first permanent molars starting from the eighth month of pregnancy and the mineralization of the incisors starting from third month after birth. Therefore, P deficit in this vulnerable phase can have undesirable effects on tooth maturation and mineralization.

**Figure 1:**
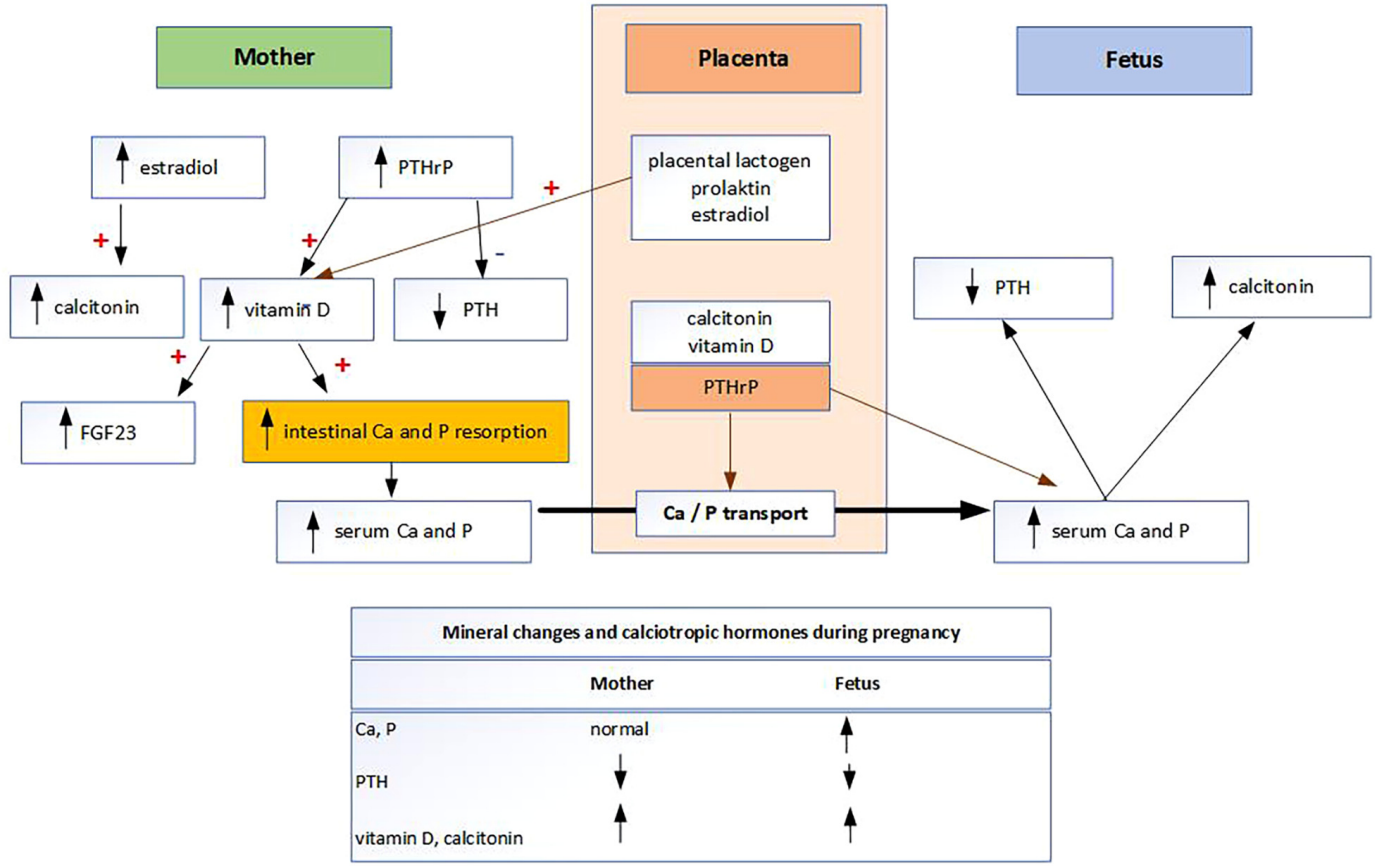
Mineral metabolism during pregnancy..

The main mechanisms by which Ca and P homeostasis are regulated to meet increased demand during pregnancy is a doubling of maternal intestinal Ca and P absorption (Figure 1). Mineral metabolism of fetus is differently regulated than in adults (4, 5). Fetal mineral metabolism and mineral transplacental transport are regulated by the placenta (4, 5). The placenta is the main source of minerals rather than the kidneys, intestines, or skeleton of the fetus (5). Mineral transplacental transport is regulated by parathyroid hormone-related protein (PTHrP) and possibly by parathyroid hormone (PTH), but not by fibroblast growth factor 23 (FGF23), calcitonin, calcitriol, or the sex steroids (6). As PTH levels are physiologically very low during pregnancy, it is likely that the main hormone regulating transplacental Ca and P transport is PTHrP produced by the placenta (7). To meet their high mineral requirements, the fetus maintains high Ca and P levels through active, sodium-dependent Ca and P transport across the placenta against a concentration gradient (8). As 80% of transplacental Ca and P transport occurs in the third trimester, it follows that preterm infants suffer from Ca and P deficiency (4, 8). Known Ca and P deficiencies are routinely supplemented in preterm infants according to the European Society of Pediatric Gastroenterology, Hematology and Nutrition (ESPGHAN) recommendations (9). It is noteworthy that the recommended dose for Ca and P supplementation in preterm infants has doubled in the last 12 years (9). However, P and Ca are not routinely measured in term infants and therefore not substituted. Other groups of newborns in which P deficiency due to placental insufficiency are expected are “small-for-gestational age babies” and infants with “intrauterine growth restriction” (4). While the mechanism of Ca homeostasis in pregnancy is known, the mechanisms of P homeostasis and transplacental P transport at the molecular level are not yet fully understood (10). P is responsible for several functions in the human body. One of these is the development and mineralization of all structural components of the teeth, as P is an essential component of enamel, dentin, cementum, and alveolar bone (7). In neonates, the total body P is about 16 g and, similar to Ca, about 80% of P is transported in fetuses during the last trimester of pregnancy at a rate of 75 mg/kg weight/day (7). About 85% of total body P is found in bone, primarily as hydroxyapatite and as complex amorphous forms of bone crystals (7). In contrast to Ca, 15% of P is widely distributed in non-skeletal tissues, in inorganic forms and as a component of structural macromolecules (7).

Fetal and neonatal availability of P is essential for the mineralization process, and thus, the direct link between hypophosphatemia and dental alterations is obvious (11). As fetal and neonatal bone mineralization is a priority, P deficiency could be compensated for by impaired tooth mineralization (11–13). Maternal and subsequent fetal P deficiency could therefore affect tooth mineralization.

**Figure 2:**
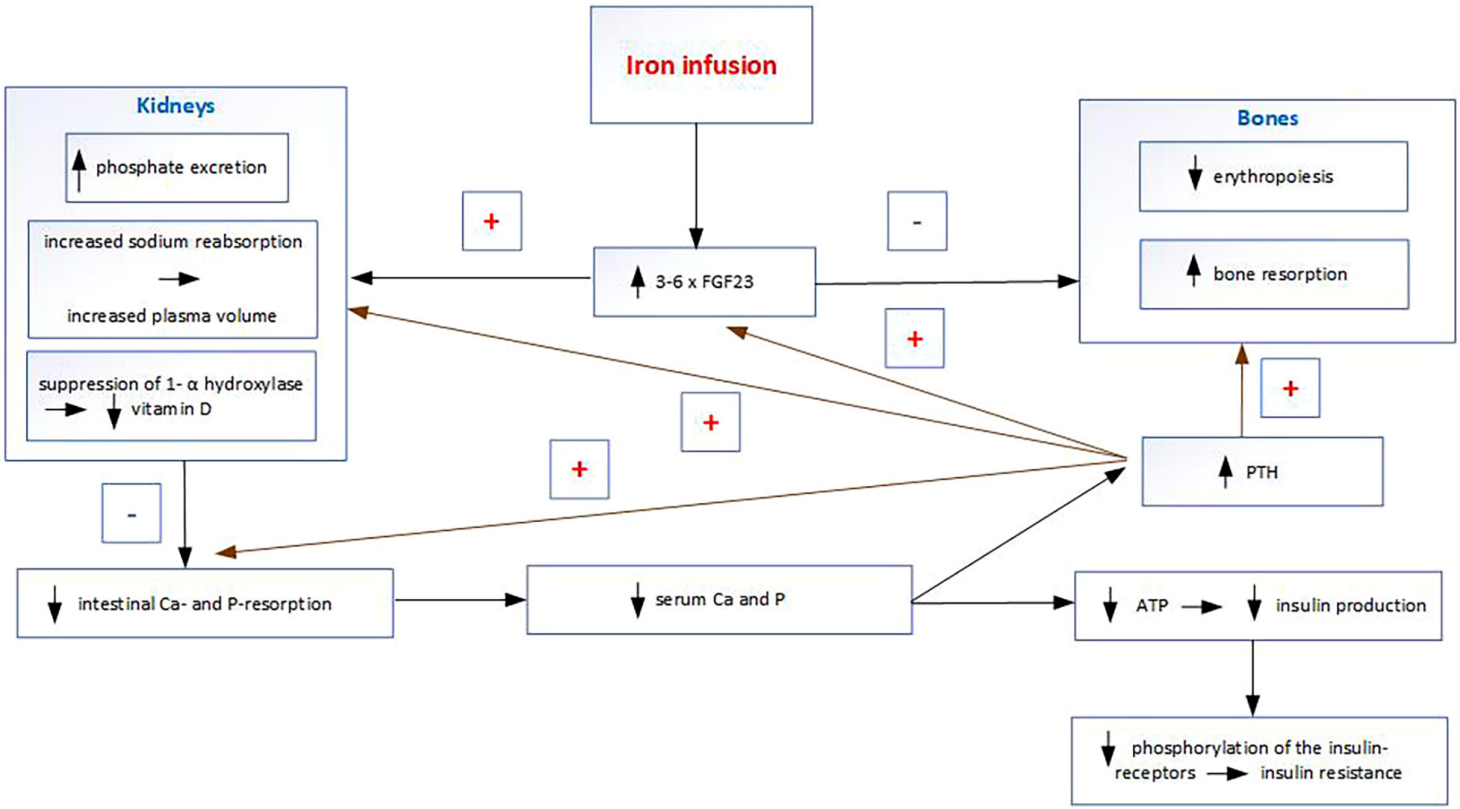
Maternal mineral changes after some iron infusions.

Some intravenous iron preparations, particularly ferric carboxymaltose, cause increased FGF23 concentrations by inhibiting its cleavage. FGF23 inhibits renal reabsorption of P in the proximal tubule, causing phosphaturia and subsequent hypophosphatemia (Figure 2). FGF23 also inhibits the activation of 25-hydroxyvitamin D (25OHD) to 1,25-dihydroxyvitamin D (14). Decreased calcitriol leads to reduced intestinal Ca and P absorption, which causes hypocalcemia. Hypocalcemia induces an increase in PTH, which leads to phosphaturia and prolongs hypophosphatemia after the FGF23 increase has returned to normal (15). It follows that some intravenous iron infusions during pregnancy result in a condition that is the opposite to the physiological changes in pregnancy. Although FGF23 does not regulate and therefore does not affect transplacental Ca and P transport, its non-physiological increase in the mother induces hypophosphatemia, hypocalcemia, hypovitaminosis D, and secondary hyperparathyroidism. The non-physiological increase in PTH (which is normally very low in pregnancy) after intravenous iron infusion may directly affect Ca and P transport across the placenta, and indirectly through PTHrP. The consequent deficiencies of P, Ca, and calcitriol in the fetus may then lead to insufficient mineralization. Other effects of increased FGF23 include stimulation of sodium reabsorption in the kidneys and increased plasma volume and thus an increase in blood pressure; inhibition of erythropoiesis leading to anemia (16) and induction of hyperglycemia (17).

There are two important issues concerning hypophosphatemia after iron infusions. The first is the long duration of hypophosphatemia after iron infusions as it lasts weeks or months (18–20). The second important issue is the vulnerable period of their administration as iron infusions are usually administered at the end of the second and the beginning of the third trimester (about 80% of iron infusions), when Ca and P transport across the placenta is maximal. On the other side, critical appraisal of studies addressing the management of iron deficiency anemia in pregnancy leads to the conclusion that the increase in intravenous iron use in the last 10 years has been driven by marketing and convenience rather than evidence of clinical benefit (21). For instance, data from Australia show that the number of women of reproductive age receiving iron infusion has more than doubled between 2014 and 2017 (21).

## Discussion

As there is an association between Ca and P deficiency and dental disruptions in X-linked hypophosphatemia rickets (XLH) (11, 22, 23), we hypothesize a possible similar association between maternal hypophosphatemia after some iron infusions and dental dysplasia in children. The clinical dental findings in XLH cases are very diverse and hypophosphatemia is the cause of multiple alterations in the dentin and enamel in both the primary and permanent teeth (11, 22, 23). The dentin shows various structural abnormalities resulting in the dysplastic and hypomineralized circumpulpal dentin with large areas of interglobular dentin and reduced thickness of the dentin (22). Enamel hypoplasia and thin enamel layer is significantly more common, but generally does not seem to be the predominant sign in XLH (11). Odontogenic abscesses and/or fistulas in caries-free teeth in both the primary and permanent teeth are the most common finding in XLH cases (11, 22). The most frequently affected teeth are incisors and canines followed by molars and premolars (11). Although dental abnormalities are very common in XLH, not all persons with XLH are affected by the clinical dental hard tissue defects, which may point to the existence of varying XLH subtypes (22).

The oral conditions of an individual are the result of various factors, such as the subject`s genotype, perinatal influences, nutritious diet, oral hygiene habits and other lifestyle habits. Hypoplastic and hypomineralized teeth derive from disturbances in tissue matrices formation and/or mineralization during odontogenesis. These teeth are more porous, undergo posteruptive tissue breakdown, and are predisposed to caries (24). Furthermore, when the incisors are affected, the associated opacities on these anterior teeth may result in cosmetic and psychosocial issues (13, 24). The etiology is still unknown, probably multifactorial and there have been over 100 different sources identified in causing hypoplastic and hypomineralized teeth (13, 24). The most common teeth to be affected are the second primary molars and the first permanent molars. Management of dental dysplasia can be challenging, and treatment approaches vary widely in different countries and in specialist and non-specialist services (24). The potential burden relating to dental dysplasia, from both an individual and a population perspective, is well recognized and continues to stimulate wide public and professional interest.

As the long-term clinical impact of maternal hypophosphatemia on the fetus has not yet been investigated, studies are required to examine the effects of maternal hypophosphatemia on the fetus. Downplaying this problem may have long-lasting health-psychosocial consequences for individuals and may represent a major socioeconomic burden. Close cooperation between obstetricians, pediatric dentists, and pediatricians is essential to study the effect of hypophosphatemia induced by intravenous iron infusion on the primary and permanent tooth maturation and mineralization, growth, and development in children.

## Data Availability

The original contributions presented in the study are included in the article/Supplementary Material, further inquiries can be directed to the corresponding author.
